# Attention-Guided Sample-Based Feature Enhancement Network for Crowded Pedestrian Detection Using Vision Sensors

**DOI:** 10.3390/s24196350

**Published:** 2024-09-30

**Authors:** Shuyuan Tang, Yiqing Zhou, Jintao Li, Chang Liu, Jinglin Shi

**Affiliations:** 1 State Key Laboratory of Processors, Institute of Computing Technology, Chinese Academy of Sciences (CAS), Beijing 100190, China; zhouyiqing@ict.ac.cn (Y.Z.); jtli@ict.ac.cn (J.L.); liuchang@ict.ac.cn (C.L.); sjl@ict.ac.cn (J.S.); 2 Institute of Computing Technology, Chinese Academy of Sciences, Beijing 100190, China; 3 University of Chinese Academy of Sciences, Beijing 100049, China; 4 Beijing Key Laboratory of Mobile Computing and Pervasive Device, Beijing 100190, China

**Keywords:** pedestrian detection, computer vision, attention-guided feature enhancement, convolutional neural network

## Abstract

Occlusion presents a major obstacle in the development of pedestrian detection technologies utilizing computer vision. This challenge includes both inter-class occlusion caused by environmental objects obscuring pedestrians, and intra-class occlusion resulting from interactions between pedestrians. In complex and variable urban settings, these compounded occlusion patterns critically limit the efficacy of both one-stage and two-stage pedestrian detectors, leading to suboptimal detection performance. To address this, we introduce a novel architecture termed the Attention-Guided Feature Enhancement Network (AGFEN), designed within the deep convolutional neural network framework. AGFEN improves the semantic information of high-level features by mapping it onto low-level feature details through sampling, creating an effect comparable to mask modulation. This technique enhances both channel-level and spatial-level features concurrently without incurring additional annotation costs. Furthermore, we transition from a traditional one-to-one correspondence between proposals and predictions to a one-to-multiple paradigm, facilitating non-maximum suppression using the prediction set as the fundamental unit. Additionally, we integrate these methodologies by aggregating local features between regions of interest (RoI) through the reuse of classification weights, effectively mitigating false positives. Our experimental evaluations on three widely used datasets demonstrate that AGFEN achieves a 2.38% improvement over the baseline detector on the CrowdHuman dataset, underscoring its effectiveness and potential for advancing pedestrian detection technologies.

## 1. Introduction

**Figure 1:**
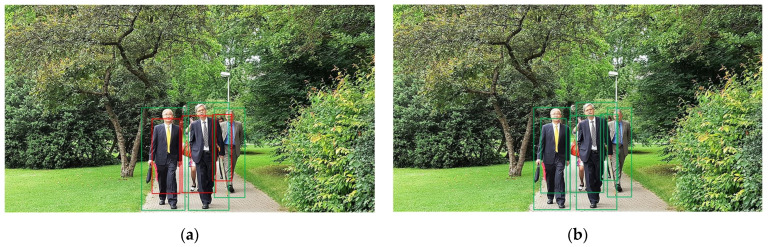
Pedestrian detection results in a crowded scenario. The green boxes indicate correct predictions, and the red boxes indicate missed predictions. (**a**) Baseline results; (**b**) our results.

Pedestrian detection [1,2,3] is a crucial technology in intelligent transportation systems [4,5,6], with numerous practical applications in advanced driver assistance systems, autonomous driving, area monitoring, human–computer interaction, and other fields [7]. In recent years, the development of deep convolutional neural networks (CNNs) has driven rapid advancements in computer vision, evolving from object recognition [8] to general object detection [9,10,11], and now making significant progress in occluded pedestrian detection [12,13,14,15,16,17,18,19,20,21]. While most pedestrian detection algorithms perform well in non-occluded and lightly occluded scenarios, state-of-the-art algorithms still struggle in heavily occluded scenes. Therefore, unlike general object detection techniques, pedestrian detection algorithms must focus on addressing the challenges posed by occluded pedestrians. To illustrate the impact of occlusion on detection, Figure 1 shows the performance of a pedestrian detector with a feature pyramid network (FPN) [22] based on the Faster R-CNN [9] architecture when applied to a heavily occluded scene. The higher missed detection rate is attributed to significant intra-class occlusion among pedestrians, resulting in some pedestrians only having small regions (such as heads or limbs) exposed in the camera’s field of view, which lowers the confidence for these targets. Consequently, improving the ability of CNNs to identify visible features has become a research hotspot in the field of pedestrian detection.

Holistic detection [12,13] is the current mainstream strategy for pedestrian detection, assuming that pedestrians are fully visible and training networks with full-body annotations. However, it is inevitable that pedestrians will be partially or heavily occluded, making the partial pedestrian features provided during network training with the holistic detection strategy incomplete. Such fragmentary features are likely to lead to inaccuracies in the final model. Additionally, the full-body detection window may be mostly filled with obstructions (background), further weakening the accuracy of the pedestrian detector.

Eager to further improve the performance of pedestrian detectors, some researchers combine multiple detectors that recognize specific parts of pedestrians to address occlusion [23,24,25,26]. This approach decomposes a pedestrian model into multiple sub-models (such as head, hands, feet, etc.) and then trains each sub-model separately to describe each part of the pedestrian. Ultimately, all sub-models are integrated to obtain a complete pedestrian detection framework. However, the improvement in the accuracy of such pedestrian detection algorithms comes at the cost of increased computational complexity. Additionally, these manually configured subcomponents may be suboptimal.

Unlike the strategy of combining multiple sub-models to solve the occlusion problem, other researchers have focused on the feedback effect of the visible parts of pedestrians on the full body. More specifically, these networks [15,27] integrate various attention modules within the standard pedestrian detection framework, aiming to make the computer focus on visible features or highlight visible regions while suppressing occluded parts, thereby achieving a higher detection rate. However, current attention-based pedestrian detection networks [14,16] often require an additional visible region training branch. This branch produces RoI features for the visible body under supervised conditions and then integrates them with RoI features generated by the full body branch. The essence of the fusion process is to re-modulate the RoI features of the full body branch using those of the visible body branch. Additionally, some methods, such as [14], enhance the representation of RoIs for the visible body by converting them into masks. Consequently, utilizing masks to emphasize pedestrian-visible region features constitutes an efficient and accurate solution. Finally, the confidence score for integrated RoIs is calculated by the classification network. However, this method has two shortcomings: First, obtaining annotations for visible regions, especially dense pixel segmentation annotations, is challenging. Secondly, the fusion method of the two RoI features needs to be finely designed, increasing the detector’s complexity.

Furthermore, when pedestrians are heavily occluded, the number of pixels belonging to them is minimal, posing a significant challenge for current CNN-based pedestrian detectors. Small-scale pedestrians have very little feature information, which becomes even more incomplete after multiple downsamplings. For the task of small-scale pedestrian detection, it is natural to consider improving the detector’s accuracy by fusing the position information of low-level feature maps with the semantic information of high-level feature maps. Liu [28] initially attempted to use multi-layer feature maps to detect objects of different scales. However, due to the small receptive field and insufficient semantic information, this method’s detection ability for small-scale objects is still not ideal. Fortunately, the Feature Pyramid Network (FPN) [22] adopts a top-down path and lateral connections to compensate for these shortcomings, truly realizing the coexistence of high-resolution and high-level semantic information. This network is easy to integrate into various object detection frameworks, significantly improving the detection rate of each network [29,30,31]. Therefore, this network is included in the deep architecture of this paper. Although FPN enriches pedestrian features to a certain extent, its top-down fusion method is somewhat simplistic, only transmitting high-layer semantic information to lower layers through element-wise addition without fully capturing the texture information of lower layers. Consequently, the performance of FPN in crowded scenarios is not optimal, leaving room for improvement. In this work, we constructed an FPN-like network based on the idea of FPN, operating from top to bottom, first extracting high-layer channel-level semantic information and then using this information to guide sampling layer by layer, thereby mapping channel-level semantic information onto low-layer spatial-level information to enhance pedestrian detail features.

Additionally, a two-stage pedestrian detector with FPN first generates a large number of target proposals with high repetition rates using the Region Proposal Network (RPN) and then removes duplicates using Non-Maximum Suppression (NMS) in the post-processing stage. The purpose of NMS is to ensure that one proposal corresponds to one prediction, which inevitably leads to the removal of certain true positives. As shown in Figure 1a, the head of the pedestrian on the right is likely recognized by the detector, but due to occlusion, the two pedestrians share most of the same features, resulting in significant overlap between the two predictions. However, the confidence score of the pedestrian behind is lower than that of the pedestrian in front, leading to the prediction for the pedestrian behind being mistakenly deleted by NMS. There are already several improvement strategies for this issue. Refs. [32,33] add a re-scoring step to traditional NMS, which reduces the scores of overlapping boxes instead of directly setting them to zero, to retain true positives. Ref. [34] builds a neural network that runs on scored detection to learn and adapt to data distribution, thereby overcoming the shortcomings of naive NMS. However, the above methods either perform poorly in heavily occluded scenarios or are difficult to implement and integrate into detectors. Therefore, ref. [35] re-examined the intrinsic relationship between proposals and predictions and designed a Set NMS method to remove duplicates while retaining true positives as much as possible. More specifically, Set NMS binds a proposal with multiple overlapping predictions to form a prediction set and then compares and removes duplicates between each prediction set. Due to the effectiveness and low cost of Set NMS in handling crowded scenarios, we will use it instead of naive NMS to further improve the detector’s performance.

To sum up, the main contributions of this paper are as follows:

(1) We develop an FPN-like network called the AGFEN, which connects the feature maps of each layer through a top-down path. AGFEN extracts high-layer channel-level semantic features and leverages them to guide low-layer features for sampling, resulting in the simultaneous enhancement of pedestrian semantic information and detailed information. AGFEN achieves similar effects to mask-based feature re-modulation approaches without requiring an additional attention branch.
(2) We combine the Set NMS method with the constructed pedestrian detection network in a reasonable manner, enabling the detector to exhibit stronger competitiveness in crowded scenarios.
(3) To enhance collaboration with Set NMS and minimize false positives, we propose a RoI feature aggregate (RoI-A) approach that reuses the classifier’s weights to aggregate local features of each RoI, thereby mitigating object confusion. Experimental results validate the exceptional pedestrian detection capability achieved through this combination strategy.
(4) We conduct extensive experiments on three commonly used datasets, confirming that the designed pedestrian detector can effectively handle detection tasks in crowded scenarios, achieving a gain of 2.38% relative to the baseline FPN.

The rest of this paper is arranged as follows: Section 2 describes the current state of the art in pedestrian detection and occlusion handling. In Section 3, the specific details of AGFEN, RoI-A, and Set NMS are presented. In Section 4, we experimentally verify the performance of the proposed network and conclude the paper in Section 5.

## 2. Related Work

Pedestrian detection research has made significant progress over the past decade, with implementation methods continually evolving and deep learning-based approaches now dominating the field. The main challenge in pedestrian detection is that occlusion significantly reduces the amount of information available to the detector, leading to missed detections. Therefore, this section will first briefly introduce deep learning-based pedestrian detection methods and then review current popular strategies for handling occlusion.

### 2.1. Pedestrian Detection

Currently, many effective pedestrian detection methods are built on convolutional neural network models. In generic object detection architectures, the process of generating object bounding boxes and refining them reflects whether the detection is two-stage or single-stage, and the same applies to pedestrian detection. Both single-stage and two-stage pedestrian detectors typically rely on anchor box-based methods, which are effective for identifying potential regions where pedestrians might be located. The method involves using anchor points as centers and defining multiple prior boxes with different aspect ratios to represent the initial states of candidate objects. These boxes are then classified and regressioned. This detection process can be repeated, with the result of anchor box regression being used as the new anchor box, followed by further classification and regression, thereby improving accuracy. Thus, whether or not this operation is repeated is a key distinction between single-stage and two-stage detectors. Since the introduction of the anchor box, it has become an indispensable part of high-precision object detection (e.g., Faster R-CNN [9], SSD [28], YOLO series [36,37,38], etc.). Theoretical analysis and experimental results have shown that two-stage pedestrian detectors [14,15,17,18] often achieve higher precision, whereas the real-time performance of single-stage strategies [12,13,39] is typically achieved at the expense of accuracy.

Single-stage detectors, such as SSD and the YOLO series, do not require generating region proposals but directly generate object class probabilities and position coordinates. Because single-stage detectors offer a one-step process, some researchers argue that they are more aligned with industry demands for real-time object detection algorithms and are easier to implement. Consequently, many single-stage pedestrian detection algorithms have been designed based on this concept. For instance, Ren [12] designed an end-to-end object detection network by introducing a recurrent rolling convolution architecture on multi-scale feature maps. Cong [13] addressed the problem of pedestrian occlusion in crowded scenes by using a repulsion loss function to optimize YOLOv3’s performance. Ref. [39] proposed an asymptotic localization fitting (ALF) module and a bottleneck block; the ALF gradually refines the default anchor boxes in SSD, while the bottleneck block optimizes the predictor by integrating residual learning and multi-scale context encoding. The integration of these modules in a single-stage detection architecture strikes a good balance between accuracy and real-time performance. In sparse scenes, some single-stage pedestrian detectors can even outperform two-stage networks in detecting large objects.

As for current research, Faster R-CNN [9] truly integrates feature extraction, candidate region generation, object classification, and bounding box regression into a deep structure, significantly enhancing its overall performance. In terms of detection speed, the RPN shares underlying convolutional features with Fast R-CNN and incorporates candidate sets into end-to-end learning, greatly improving the efficiency of candidate box generation. The exceptional performance of Faster R-CNN has made it a milestone algorithm in pedestrian detection and a typical two-stage pedestrian detector. As a result, Faster R-CNN is widely used as a benchmark network in pedestrian detection, and many pedestrian detectors based on Faster R-CNN have achieved excellent results. For example, Zou [17] added a multitask correction attention module to Faster R-CNN to generate masks for multiple visible parts of pedestrians and correct false regions, enhancing the correlation of pedestrian body features. Wang et al. [18] improved RPN by constructing a new pedestrian area generation network and using a soft cascaded decision tree to combine features of various resolutions and layers to handle classification problems. Given the excellent detection accuracy of the two-stage pipeline, this paper builds on this foundation.

### 2.2. Schemes for Handling Occlusion

Part-based models are currently a popular strategy [23,24,25,26,40] that divides and integrates various parts of pedestrians to handle occlusion. Noh et al. [23] proposed training local detectors individually, then comprehensively considering the scores of all local detectors to reduce the miss rate. Zhang [24] designed a novel aggregation loss function and an occlusion-aware RoI pooling operation to improve overall detection accuracy by predicting various parts of the human body. Ref. [25] proposed DeepParts, composed of multiple part detectors, which can be trained even with data lacking part annotations and can also calibrate positive proposals that differ significantly from the ground truth. Ref. [26] utilized a strategy of sharing a set of decision trees among multiple part detectors, improving the correlation between detectors and effectively reducing the computational complexity of the overall network. This multi-detector joint learning method outperforms approaches where each detector learns separately. Ref. [40] constructed a reciprocating feature adaptation and iterative pedestrian detection network called CircleNet, which uses multiple circle structures to learn different features of pedestrians.

Another approach to dealing with occlusion is to introduce an attention mechanism. These methods commonly exploit the visible regions of pedestrians to generate attention feature maps, which guide the detector to learn the visible features of pedestrians. Wang [41] focused on dense crowd scenes and proposed a repulsion loss between pedestrians, ensuring the current object’s prediction is as far from others as possible. Ref. [14] integrated a novel mask-guided module into a standard pedestrian detector and proposed an occlusion-sensitive hard example mining method, leading to significant improvements in detection performance. Similar to [14], Refs. [16,27] introduced additional visible region branches to enable the network to learn the visible features of pedestrians under the supervision of visible region annotations. However, some researchers argue that attention-based (or saliency-based) methods are unreliable because detection networks struggle to distinguish attention regions of positives from those of negatives. Consequently, Ref. [19] intensified multi-channel features by sharing classifier weights in convolutional layers to obtain higher-level semantic information. The enhanced multi-channel features are superimposed to form a pedestrian self-activation map, selectively highlighting the visible parts of pedestrians. Although the self-activation map undergoes pixel-level and region-level corrections, its performance in crowded scenes still lags behind that of attention mechanism-based methods. Therefore, we continue to focus on the application of attention mechanisms to enhance feature representation. Ref. [15] argued that different channels activate different body parts. Drawing inspiration from this study but differing from approaches that add an attention branch [14,15,16,27], we construct a top-down feature enhancement network that maps channel-level activations.

## 3. Improvement Proposal

In this work, we focus primarily on pedestrians who are either obscured or appear small in the camera’s field of view. It is evident that these pedestrians are challenging to identify, necessitating targeted solutions. Inspired by the FPN’s ability to extract multi-scale features, we designed an attention-guided deterministic sampling module with an FPN-like structure to capture detailed pedestrian features. We then developed a novel pedestrian detector based on the standard Faster R-CNN framework. Specifically, the semantic information from the high-level feature map guides the high-resolution feature map to sample specific local regions, thereby directing the computer’s attention to the visible parts of occluded pedestrians. Simultaneously, the second-stage classifier’s weights are employed to aggregate the local features of RoIs, enabling the network to distinguish easily confused samples and thereby improving detection performance.

**Figure 2:**
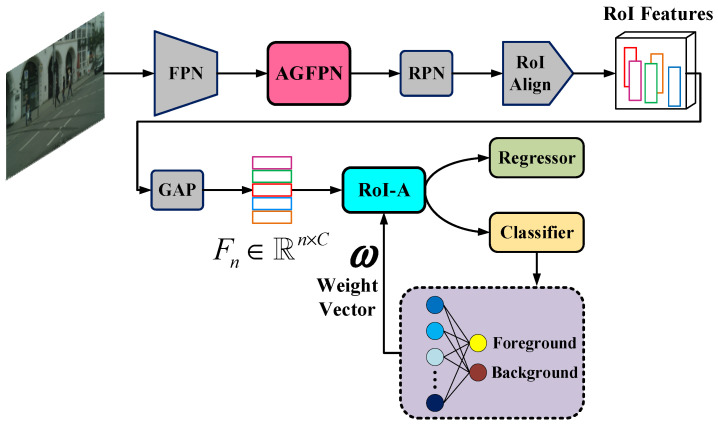
An overall architecture of the attention-guided feature enhancement network (AGFEN). It is a faster R-CNN with a ResNet-50+FPN as its backbone, also with an additional attention-guided feature pyramid network (AGFPN) and a RoI features aggregation (RoI-A) operation. AGFEN uses high-level semantic features to enhance the texture of low-level features and strengthen the boundary information between pedestrians and background so as to achieve the purpose of highlighting pedestrians while suppressing background. In the meantime, the detection network also makes full use of the weight of the classifier to aggregate the features of each RoI, thereby improving the representation of RoIs.

The overall architecture of the novel pedestrian detector is illustrated in Figure 2. Although ResNet has more layers than VGG-16 and VGG-19, we replace the fully connected layer with global average pooling (GAP), allowing the model to occupy relatively less memory while effectively preventing overfitting. Therefore, we adopt ResNet-50 with FPN as the backbone to extract pedestrian features and then feed the resulting feature map into the FPN-like multi-scale feature extraction network, aiming to emphasize partial features by capturing spatial details. Finally, after completing the RoI-A operation using shared classifier weights, the classification and regression tasks are performed based on the aggregated features.

### 3.1. Attention-Guided Feature Pyramid Network

Figure 2 clearly illustrates the position of the Attention-Guided Feature Pyramid Network (AGFPN) within the overall pedestrian detection pipeline, with its structure and functions detailed below. As is well known, the backbone network uses max pooling layers to capture the feature textures of pedestrians while reducing interference from non-target objects such as vehicles, trees, and buildings. Although max pooling ensures insensitivity to feature changes caused by local pedestrian displacement, features of pedestrians that are distant from the camera or heavily occluded may vanish after downsampling and max pooling. This means that relying solely on the features extracted from the backbone cannot achieve satisfactory detection accuracy. While embedding an FPN can effectively enhance detection capability, the performance in crowded scenarios remains suboptimal. Therefore, we propose an AGFPN that not only preserves global contextual relationships but also highlights local features. Similar to the FPN, it operates through top-down and horizontal connections, aiming to fully integrate high-level semantic information and low-level visual information while compensating for some of the errors introduced by score-level fusion methods.

**Figure 3:**
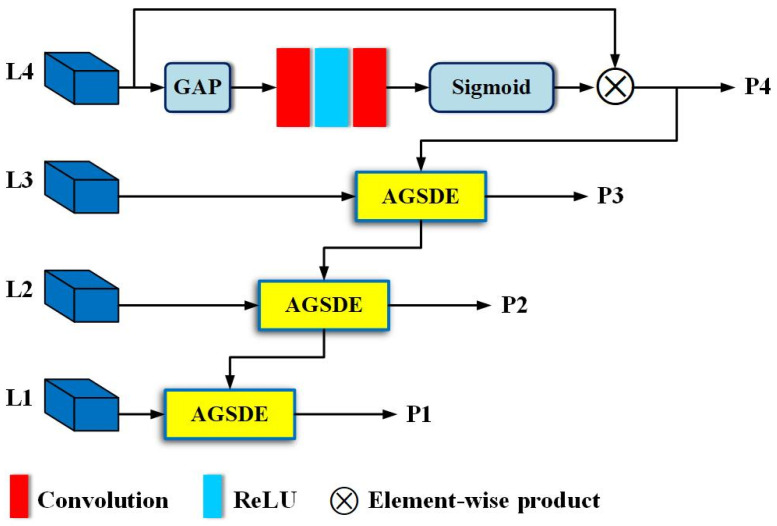
The structure of AGFPN. This is the FPN-like network, which is used to add attention information to high-resolution visual information. This process is implemented under the guidance of high-level semantic information.

Specifically, as illustrated in Figure 3, we perform GAP on the output feature map of the fourth convolutional layer, trying to retain the background information that contributes to classification in the deep feature map, and obtain a channel-wise statistic descriptor A. Given the output of the fourth convolutional layer is $P4\in {\mathbb{R}}^{C\times H\times W}$, then $A\in {\mathbb{R}}^{C\times 1\times 1}$. Next, based on descriptor A, we explore the interaction relationships between multiple channels that constitute the high-level semantic feature map. The output of GAP is followed by a convolution layer, an activation function ReLU, and another convolution layer, and then a Sigmoid function is used to obtain the correlation between channels. Thus, a new channel statistical descriptor, $A^{\prime }\in {\mathbb{R}}^{C\times 1\times 1}$, is yielded from the above process. Finally, $A^{\prime }$ is fed back to L4, and both are conducted element-wise to generate a channel attention feature map P4.

Researchers suggest that the activation of different body parts is influenced by various channel features, and the relationship between cross-channel feature mapping can improve the detector’s discrimination [15,42,43]. Specifically, P4 utilizes squeeze and excitation to establish intrinsic relationships within each channel and uses these relationships as weights to enhance high-layer features extracted by the backbone. As a result, a channel-wise enhanced version with high-level semantic information is generated. However, relying solely on the relationship between channels in high-layer features for detector optimization may yield limited or even negative results. This is because high-level semantic information has the potential to obscure target object details, leading to inaccurate regressions or missed detections in heavily occluded environments.

Inspired by FPN [29], we utilize the enhanced high-level semantic information as a guide for low-layer visual information, enhancing features at all levels except for L4 in a top-down manner to alleviate the problem of ambiguous detail information. Unlike FPN, AGFPN does not directly adopt the operation mode of upsampling and element-wise addition, which may avoid conflicts between high-level semantic features and low-level visual features at certain pixel points after upsampling, thus preventing feature degradation. Figure 3 illustrates, on the other hand, that P4 is not directly used to guide L1 and L2 to pay attention to the special details within them. This decision aligns with the original intention of developing AGFPN. We aim to preserve contextual information as much as possible and tolerate certain errors caused by layer-by-layer guidance. This approach is particularly applicable in heavily occluded scenes, where contextual relationships can enhance the detector’s understanding of complex scenes, such as pedestrians typically appearing on roads rather than in the sky or rivers. Therefore, sampling solely from the current layer to guide the previous layer in strengthening target object details is an intuitively effective scheme.

**Figure 4:**
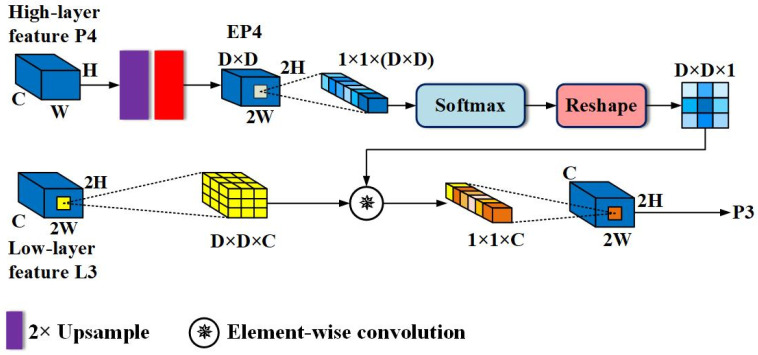
The structure of the attention-guided sampling-based details enhancement module. Note that for simplicity, the diagram uses L3 link as an example to show AGSDE in detail. The AGSDE on the rest of the links follows exactly how this diagram operates.

Attention-Guided Sampling-Based Detail Enhancement Module: as mentioned earlier, both semantic information and detailed information are indispensable for the accurate classification and regression of pedestrians. Semantic information ensures the correct classification of pixels, while detail information can determine which pixels belong to pedestrians, providing relatively accurate information for the detector to learn occlusion patterns. Thus, enhancing the detail features of pedestrians with semantic information can boost detector recognition of hard examples. This idea is similar to a pedestrian detector trained using external visible region annotations, which essentially focuses on learning the features of visible parts of the pedestrian and then uses the training results to enhance the full body features or implement score-level fusion of two branches. In contrast, we directly utilize the existing cross-channel attention feature P4 to produce similar effects without additional attention branches. To achieve this, a module called Attention-Guided Sampling-Based Details Enhancement (AGSDE) was constructed. Figure 4 shows AGSDE in detail.

For the sake of simplicity, only the AGSDE module on the L3 link will be explained. Firstly, perform a bilinear upsampling with a factor of 2 on the enhanced high-layer feature P4 to obtain a feature map that matches the scale of low-level feature L3. Next, pass it through a convolutional layer of size 3 × 3, with the aim of compressing the number of channels to (D × D), where (D × D) also represents a region in L3 that is about to be sampled. Now, P4 has transformed to EP4 with dimensions of (D × D) × 2H × 2W. Then, take any point (*i*, *j*) from EP4 and perform Softmax normalization on this point in channel dimension, and obtain a vector of the contribution degree of each channel at this point, whose size is (D × D) × 1 × 1. This vector is then reshaped to a two-dimensional plane with size D × D × 1, and the plane is treated as the convolution kernel. The kernel will convolve with the region centered on (*i*, *j*) in L3, and the convolution operation is as follows:

(1) $$P3_{\left(i,j\right)}=\sum_{w=-r}^{r}\sum_{h=-r}^{r}L3_{\left(i+w,j+h\right)}K_{\left(w,h\right)}$$

where $r=\left\lfloor D/2\right\rfloor$, K represents the convolution kernel generated from any point in EP4. By executing Formula (1) for each point on L3, feature P3, guided and enhanced by a high-layer semantic feature, is obtained. Similarly, P3 can guide L2 to yield P2, and P2 can guide L1 to yield P1. As such, the structure of the entire AGFP is a standard pyramid shape.

After embedding the AGSDE module, the output will occur in the following three cases: (i) When sampling points inside a pedestrian (such as a pixel on the head), a certain channel will exhibit a higher value. As a result, the higher value will be reshaped into the center of a two-dimensional plane, and the further away from the center, the smaller the corresponding channel value. Thus, the pixel is significantly enhanced after convolution. (ii) When sampling the boundary between pedestrians and background, these points will be affected by nearby points, so the boundary may be slightly enhanced or suppressed. (iii) When sampling points outside of pedestrians, the channel reflecting the background will dominate. Meanwhile, the values of background and its surroundings are small, and the convolutional result is also small, thus the background is suppressed relative to pedestrians. After modulating the features of the backbone in this way, there is abundant contextual information to assist prediction. For boundary and small-scale pedestrians, it will also show relative stronger activation.

### 3.2. RoI Aggregation

After the aforementioned work is completed, generally, the enhanced features are used to generate a large number of proposals through RPN, and then R-CNN is used to achieve fine classification and regression for each proposal. However, intra-class occlusion results in a narrow gap between individuals; the detector is likely to regard multiple overlapping pedestrians as a single target. Meanwhile, AGFPN activates pedestrian features based on channel-level attention; certain background regions similar to pedestrian features will also exhibit high activation. In addition, we have adjusted the way instances are generated and removed (details will be shown in Section 3.3). There will be multiple prediction instances corresponding to one proposal, so that some false positives may be incorrectly retained, ultimately leading to an increase in detector false positives. As such, it is necessary to design solutions from the perspective of RoIs.

Considering that the inherent connections between various RoIs can reflect the local clues of each RoI, and aggregating the local information of each RoI can improve the internal features of the RoI, helping to prevent object confusion [44,45], and ultimately having the potential to further improve detection performance. More specifically, we first perform GAP on the RoI features and then use the weight vector $\omega ={\left(\omega^{1},\omega^{2},\ldots ,\omega^{C}\right)}^{T}\in {\mathbb{R}}^{C}$ of the R-CNN classifier to aggregate the local channel features of each RoI, where C represents the number of channels. As a result, a new feature $F_{A}$ to be classified is yielded, which is calculated as follows:

(2) $$Z_{A}=softmax\left(F_{n}\omega {\left(F_{n}\omega \right)}^{T}\right)$$

(3) $$F_{A}=\sigma \left(Z_{A}F_{n}\right)$$

where $F_{n}\in {\mathbb{R}}^{n\times C}$ represents a channel-level feature matrix composed of n RoI features arranged in rows, and σ represents the LeakyReLU activation function. Next, split $F_{A}$ into rows and feed these vectors to the classification and regression network. Note that ω is pre-trained and updated during training.

### 3.3. Loss Function

The loss function of the standard Faster R-CNN includes two sub-parts of RPN loss and R-CNN loss. The confidence of the RPN part adopts the binary cross-entropy loss, the confidence of the R-CNN part uses the multi-class cross-entropy loss, and their position offsets all use the Smooth-L1 loss. Therefore, the backbone can be trained end-to-end with the following loss functions:

(4) $$L_{F\_rcnn}=L_{rpn\_cls}+L_{rpn\_reg}+L_{rcnn\_cls}+L_{rcnn\_reg}$$

Although this loss function achieves unexpected results in pedestrian detection, it still suffers from a high and unsatisfactory miss rate when faced with crowded scenes. For this reason, we enhance the ability of the original Faster R-CNN to acquire pedestrian features through a series of additional modules described above and expect the detector to eliminate the interference of occlusion as much as possible. However, in an overcrowded scenario, certain proposals have very similar features and will inevitably produce highly overlapping predictions. Unfortunately, partially overlapping predictions can be discarded by NMS operations, making it impossible for the detector to generate unique detection results for each proposal. In order to preserve these positives and evolve the network towards a better direction, we adopted the scheme proposed in [31], which uses each proposal box to predict a set of highly overlapping instances and makes simple modifications to the NMS so that proposals belonging to different pedestrians but overlapping will be retained. The following will introduce the details that need to be modified to implement this plan.

#### 3.3.1. Instance Set Prediction

Instead of one proposal box $b_{i}$ corresponds to only one instance, we associate $b_{i}$ with a set of ground-truth instances $G\left(b_{i}\right)$ based on the predefined intersection-over-union (IoU) threshold:

(5) $$G\left(b_{i}\right)=\left\{g_{j}\in G\left|IoU\left(b_{i},g_{j}\right)\geq \theta \right.\right\}$$

where G is the set of all the ground-truth boxes. In this work, the number of elements in the set $G\left(b_{i}\right)$ is set to 2.

At the same time, refer to [35], an additional prediction branch is introduced into the detection framework so that each proposal box $b_{i}$ can predict different instances $\left(cls_{i}^{1},reg_{i}^{1}\right)$ and $\left(cls_{i}^{2},reg_{i}^{2}\right)$ through two detection functions, where $cls_{i}$ is the class confidence of the prediction object, and $reg_{i}$ is the relative coordinate. Therefore, the prediction set of any $b_{i}$ can be expressed as follows:

(6) $$P\left(b_{i}\right)=\left\{\left(cls_{i}^{1},reg_{i}^{1}\right),\left(cls_{i}^{2},reg_{i}^{2}\right)\right\}$$

#### 3.3.2. Formulate a Loss Function

Once obtaining the prediction set $P\left(b_{i}\right)$ and the ground-truth instance set $G\left(b_{i}\right)$, the Earth Mover’s Distance (EMD) can be used to convert the similarity measurement between the two sets into the calculation of relative distance [35,46], and then minimize the EMD function to narrow the gap between the prediction set and the ground-truth instance set. The loss function constructed based on EMD can be represented as:

(7) $$L_{EMD}=\underset{\pi \in \Pi }{\min}\sum_{k=1}^{2}\left[L_{rcnn\_cls}\left(cls_{i}^{k},g_{\pi k}\right)+L_{rcnn\_reg}\left(reg_{i}^{k},g_{\pi k}\right)\right]$$

where π represents a certain permutation of (1, 2) whose k-th item is $\pi_{k}$; $g_{\pi k}\in G\left(b_{i}\right)$ is the $\pi_{k}$-th ground-truth box; the meanings of $L_{rcnn\_cls}$ and $L_{rcnn\_reg}$ are consistent with the previous content, representing the classification loss and regression loss of R-CNN network. Note that if the number of elements in the ground-truth set is less than 2, it is feasible to manually add ground-truth instances that are labeled as background but without regression loss to meet this condition. Finally, the loss function of the entire pedestrian detection network can be expressed as follows:

(8) $$L_{AGFEN}=L_{rpn\_cls}+L_{rpn\_reg}+L_{EMD}$$

#### 3.3.3. Set NMS

In addition to modifying the correspondence between proposal boxes and ground truth instances, as well as adjusting the loss function for training, a simple fine-tuning of the naive NMS operations is also required. When standard NMS is used, predictions that overlap but belong to different individuals are still removed. After the above two steps, each proposal will produce two predictions unique to itself, and by setting a trigger for the NMS, these two predictions will not suppress each other, even if their IoU is close to 1. At this stage, suppression occurs between different proposals, meaning it acts on different prediction sets. Only if two prediction boxes originate from different prediction sets can one prediction box suppress the other. In other words, the fine-tuned NMS operates on prediction sets as the minimum unit rather than on individual predictions. This fine-tuned NMS is also referred to as the Set NMS.

### 3.4. Complexity Analysis

The loss function of the standard Faster R-CNN includes two sub-parts of RPN loss and R-CNN loss. The confidence of the RPN part adopts the binary cross-entropy loss, the confidence of the R-CNN part uses the multi-class cross-entropy loss, and their position offsets all use the Smooth-L1 loss. Therefore, the backbone can be trained end-to-end with the following loss functions:

To analyze the complexity of the proposed network, we studied the model’s floating point operations (FLOPs) under different parameters. For consistency, we represent the scale of FLOPs as GFLOPs. From the perspective of the overall pipeline, AGFEN does not alter the structure of the baseline detector. Therefore, the total GFLOPs of the proposed model equals the sum of the GFLOPs of the baseline model and the GFLOPs of the embedded modules. In AGFEN, the modules that contribute significantly to the total GFLOPs are AGFPN and RoI-A. We first analyze AGFPN. Given the smallest feature map $L4\in {\mathbb{R}}^{C\times H\times W}$ output by the FPN module, the complexity of AGFPN can be expressed as $O\left(CHWD^{2}\right)$. Since the size of the feature map is fixed by FPN, the GFLOPs of AGFPN are mainly related to the size of the sampling region D×D. It is evident that the larger the sampling region, the higher the computational complexity of the model, but the detection capability of the model also improves. For example, when selecting a 5 × 5 sampling region, the GFLOPs of the improved model increase by 22.486 compared with the baseline model.

**Figure 5:**
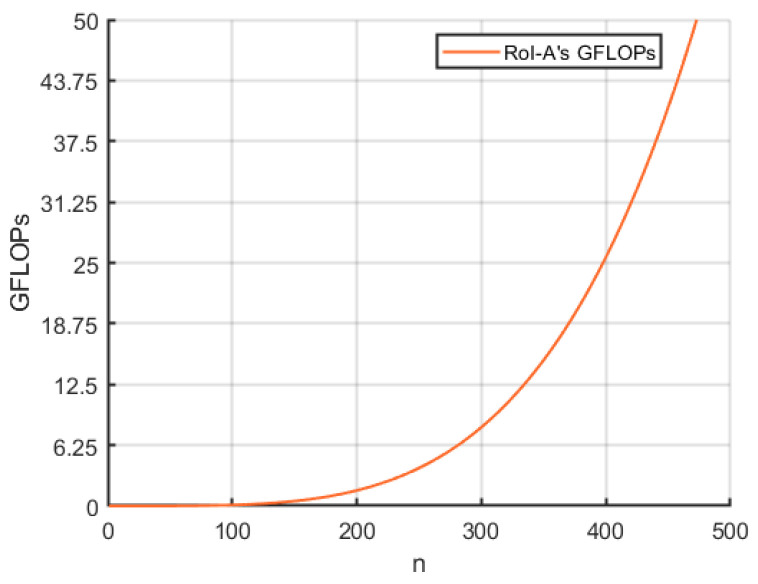
The relationship between the number of RoI feature maps and GFLOPs of ROI-A module.

Next, we analyze the computational complexity of RoI-A. As discussed in Section 3.2, RoI-A primarily involves matrix operations, and thus, its computational complexity can be expressed as $O\left(n^{4}\right)$, where n represents the number of RoI feature maps. Figure 5 illustrates the trend of GFLOPs in the RoI-A module as n varies. Both Figure 5 and mathematical analysis indicate that n has an exponential relationship with GFLOPs. In summary, when deploying AGFEN, the model’s performance and computational cost can be flexibly balanced according to practical requirements.

## 4. Results

In this section, we first describe the implementation details of the datasets, evaluation metrics, and experiments. We then evaluate the effectiveness of our modifications to the baseline network using three public and widely used pedestrian datasets. For simplicity in the subsequent discussion, AGFEN will be used to refer to the proposed pedestrian detector. Finally, we present and compare the performance of AGFEN with state-of-the-art pedestrian detectors.

### 4.1. Experimental Program

#### 4.1.1. Datasets

In order to verify the effectiveness of the network proposed in this paper, three commonly used datasets, CrowdHuman [47], CityPersons [48], and Caltech [49], are used to evaluate AGFEN. The CrowdHuman is a relatively challenging pedestrian detection dataset, which contains a large amount of data, including 15,000 training images, 5000 test images, and 4370 validation images. In the CrowdHuman dataset, the average number of pedestrians per image is as high as 23, showing a very rich occlusion pattern, and the degree of occlusion of pedestrians is high. The CityPersons dataset is a subset of CityScapes that only contains pedestrian annotations. The images in CityPersons are all recorded based on on-board cameras. The recorded scenes are multiple cities in Germany, and the image size is 2048 × 1024. It has a total of 5000 images, of which 2975 images are used for training and 500 and 1575 for validation and testing. The average number of pedestrians in an image in this dataset is 7. The image size of the Caltech dataset is 640 × 480; its image acquisition method is similar to that of CityPersons, whereas the location is relatively single and the number of people is much smaller. This dataset divides all the images into two parts, in which the six sequences from S0 to S5 are used as the training set, and the five sequences from S6 to S10 are used as the test set. Table 1 lists the representative statistics for these three datasets.

#### 4.1.2. Evaluation Metric

In order to comprehensively evaluate the performance of the proposed detector, we mainly use the following three common metrics:

- Average precision (AP) is one of the evaluation metrics of the target detection model. It is the result of smoothing the precision-recall curve and then averaging the precision value. The larger the AP, the better the detection performance.
- Log-average Miss Rate (MR^−2^) is over false positive per image (FPPI) within [10^−2^, 10^0^]. In this paper, the pedestrian detection performance of AGFEN is evaluated using the three most commonly used criteria: reasonable, heavy, and partial. Among them, the visibility ratio of reasonable sets exceeds 65%. The visibility ratio of heavy sets is ranging [0.2, 0.65]. Note that the heavy set range used by some detectors is [0, 0.65], and we will add * superscripts to the data to distinguish between the two differences. The visibility ratio of the partial set is ranging [0.65, 0.9]. The heavy set is mainly used to evaluate the performance of each pedestrian detection network under heavy occlusion conditions. For simplicity, write R for the reasonable subset and HO for the heavy subset. The lower the MR^−2^, the better the detection performance.
- Jaccard index (JI) is used to compare the similarity between the prediction set and the corresponding ground-truth set, where the prediction set is generated by a confidence threshold. The higher the JI score, the better the detection performance.

#### 4.1.3. Implementation Details

We set the baseline pedestrian detector Faster R-CNN with FPN according to the guidance provided by [22]. Additionally, the related structures and parameter changes refer to open source projects [50]. On the CrowdHuman dataset, we set the initial learning rate at 10^−5^ and decayed by a factor of 10 at the 25th and 35th epochs, respectively. A total of 50 epochs are trained. On the CityPersons dataset, we set the basic learning rate to 10^−4^ and reduce the learning rate by a factor of 10 after performing 5k iterations. The implementation details of Caltech are basically the same as those of CityPersons. First, train with a learning rate of 0.0001 for the first three epochs. Then, change the learning rate to 0.00001.

### 4.2. Ablation Study

To verify the effectiveness of the proposed method, we performed ablation experiments on three datasets.

(1) Baseline Comparison: We take the two-stage pedestrian detection network ResNet-50+FPN as the baseline and then add different modules in turn to observe the performance of the network containing different modules on the CrowdHuman dataset. Since we have modified the one-to-one correspondence between proposals and ground-truth instances in the baseline to One Proposal to Multiple Predictions (OPMP) and adjusted loss functions and a NMS operation, OPMP can be used to represent the entire modification operation. Note that in order to compare the performance of each module at one level, the network with each module added is trained using the same set of ground-truth pedestrian instances.

Table 2 reports the performance changes of pedestrian detectors after adding each module sequentially, and the best scores are shown in bold. As we changed the baseline, the new model obtained higher AP and JI scores on the CrowdHuman validation set and lower MR^−2^ scores, indicating that our changes effectively enhanced the performance of the baseline. The last row in Table 2 is the full version of the proposed network, which achieves optimal pedestrian detection performance. Compared with the baseline, the gains of the complete pedestrian detection model on AP, MR^−2^, and JI were 6.73%, 2.38%, and 3.61%, respectively. Rows 2 and 3 also clearly reflect the performance differences between FPN and AGFPN. The AGFPN replaces the score-level fusion scheme of element-wise addition by the way of guided sampling, which has great advantages in transferring high-level semantic information layer-by-layer and retaining contextual information. Especially in the AGSDE module, this sampling-based feature enhancement method can organically fuse the features of local regions, thereby enlarging the gap between the visible part of the pedestrian and the background. This is an application that maps spatial locations from channel features and is also an embodiment of the idea of semantic segmentation, which helps RPN to generate more accurate recommendations. Therefore, it is not surprising that AGFPN performs better when no other modules are introduced. It is also worth noting that RoI-A should be used with OPMP. When the model is AGFPN + RoI-A, the improvement relative to the detector without the RoI-A module is minimal; for example, the gain on the MR^−2^ metric is only 0.29%. However, when the model is AGFPN + OPMP, OPMP and AGFPN complement each other. AGFPN provides informative features for OPMP, and OPMP reduces the probability of true positives being suppressed by errors. As a result, AGFPN + OPMP can significantly improve AP, with a gain of up to 3.03%. However, because AGFPN causes multiple occlusion objects to share some RoI features in crowded scenarios, which is likely to generate false positives, the MR^−2^ score of AGFPN + OPMP is limited. Fortunately, by aggregating the local features of each RoI through the ROI-A module, this problem can be effectively improved, so that the performance of the complete detector can reach the optimal 92.52%, 40.54%, and 83.44%.

(2) Comparison With Other FPN-Like Structres: We construct an FPN-like network to enhance pedestrian feature information and then use the enhanced features to boost the performance of RPN. Similar work appears in PANet [51] and CircleNet [40]. PANet adds a bottom-up information transfer path to FPN, while CircleNet uses multiple top-down and bottom-up paths to enhance pedestrian features and share information between the up and down paths, forming a circle-like network. Based on the number of circles, the model is simply named Circle-T. Structurally, our AGFPN and PANet can be seen as CircleNet-1/2. To make a fair comparison, the FPN is used as the baseline, then the network is set up according to [40], and the input image is scaled by a factor of 1.3.

Table 3 shows that all the improvements to FPN result in a positive gain in the detector’s performance. In particular, our FPN-like structure achieved the lowest MR^−2^ scores of 10.24% and 66.48%, respectively, on two subsets of the Caltech dataset. Due to the fact that the other two structures both use element-wise addition to transmit information between layers, there is a lack of exploration and leverage channel-level semantic information, resulting in limited gain to the baseline. Although CircleNet learns different occlusion patterns by constructing multiple circles, there are significant differences in the activated features between different circles, and the lack of a more efficient circle-level feature fusion method meant that increasing the number of circles cannot yield the desired benefits. For example, on the **R** set, the performance of CircleNet-2 was reduced by 0.88% relative to CircleNet-1, while the performance of CircleNet-3 was further reduced to 14.72%. By comparison, AGFPN is a more effective approach to enhance pedestrian features through attention-guided sampling.

(3) Comparison With Other Visible-Box Attention Strategies: Our AGFPN maps channel features with high-level semantic information layer-by-layer onto features at all levels, enhancing the visible regions of pedestrians at all levels. From the final result, AGFPN is similar to methods that partially contain two training branches, such as Bi-box [27], OR-CNN [23], MGAN [14], and DMSFLM [16]. These methods were trained separately on the full body and visible regions of pedestrians, ultimately combining the results of the two parts to enhance detection performance. Therefore, AGFPN is embedded in a detector with VGG-16 as the backbone, and its results are compared with the above method.

Table 4 reports the scores of each attention strategy under the same backbone and the same scaling conditions. Note that both MGAN and DMSFLN only consider classified loss of visible body parts. All detectors with an extra visible body region branch achieved similar performance on the **R** set, but their performance on the **HO** set seriously varied due to the different design of loss functions and fusion methods. The best overall performance is MGAN, thanks to its specially designed occlusion-sensitive loss function and weak box-based segmentation annotation. Our AGFPN ranked in the middle, achieving MR^−2^ scores of 10.8% and 46.5% on the **R** and **HO** sets, respectively. Compared with the worst OR-CNN, our method achieved an improvement of 0.2% and 4.8%, respectively. However, it is worth noting that AGFPN enhances the pedestrian visible region through guided sampling, which saves the network overhead, because the cost of obtaining the visible region annotation or the more demanding dense pixel segmentation annotation is too high, and our method provides an approximate enhancement effect. Thus, AGFPN can be used as an alternative to the visible-box attention strategy when visible region annotations are missing. In addition, AGFPN can be easily embedded into other backbones.

(4) Comparison With Other NMS Strategies: We use different NMS approaches to handle the excess boxes and show the corresponding scores in Table 5. Note that the detector is a ResNet-50+FPN with AGFPN, and the threshold of the IoU is fixed at 0.5 according to [31]. Unsurprisingly, the naive NMS method scored lowest, and it roughly removed all overlapping prediction boxes. For Soft-NMS, although it improved the AP score relative to NMS, the MR^−2^ benefit in crowded scenarios was small, only 0.05%. It is clear that OPMP exhibits high AP and lower MR^−2^ compared with Soft-NMS and naive NMS. Therefore, for cost reasons, OPMP is the best choice.

(5) Further Study on RoI-A: We also explored the universality of RoI-A. Table 5 indicates that RoI-A can effectively improve the recognition capability of the network, even when paired with the naive NMS. When paired with RoI-A, the gain of Soft-NMS over the naive NMS increased from 2.4%, 0.05%, and 0.02% to 2.8%, 0.41%, and 0.29%, respectively. Experiments show that RoI-A can be combined with different NMS methods and can improve recall precision and reduce miss rates in complex, crowded scenarios.

### 4.3. State-of-the-Art Comparison on CrowdHuman Dateset

**Figure 6:**
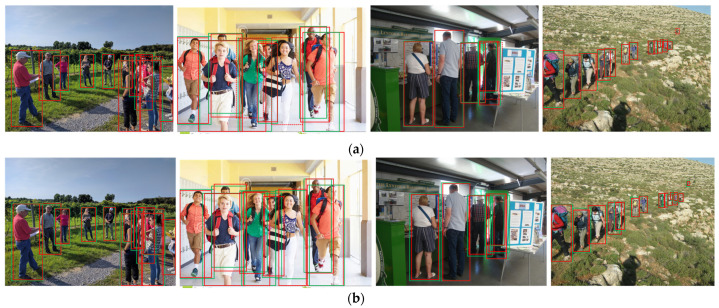
The two detectors were compared visually on CrowdHuman validation set. The solid red line boxes in these pictures represent ground truth, the solid green line boxes represent predictions of the detector, and the dotted red line boxes represent missed detections, respectively. (**a**) The recent DMSFLN; (**b**) Our AGFEN.

Table 6 reports the scores of our AGFEN, including AGFPN, RoI-A, and OPMP, against other state-of-the-art detectors on the CrowdHuman validation set. It can be clearly observed that our detector has the optimal results on the three evaluation metrics, that is, the scores are shown in bold. It is worth noting that we used the Faster R-CNN with FPN as the baseline, and it performed unexpectedly, especially under the evaluation criteria of MR^−2^, where it achieved a good second place. Most detectors did not report scores on the JI metric, but the baseline was only 1.57% different from S-RCNN [52] and performs better on the MR^−2^, achieving a gain of 1.78%. FPN only superimposes upper-layer features after up-sampling to lower-layer features and unifies the feature dimensions to facilitate the fusion of deep semantic information and shallow texture information, so as to achieve the purpose of enhancing the feature maps of each layer. The remaining detectors either improve the loss function or introduce additional attention branches, which are relatively complex to implement. However, from the perspective of the relatively important MR^−2^ index, this means of blending semantic features and texture features is very effective in enhancing the performance of the detector. For this purpose, our AGFPN superimposes semantic features onto texture features in a softer way, which not only highlights the pedestrian regions but also reinforces the boundary between pedestrians and the background. As mentioned earlier, this is similar to an enhanced feature modulated by a mask of the visible part, but AGFPN does not require additional visible region annotation. Therefore, AGFPN further increases the baseline performance while supplementing RoI-A and OPMP to minimize false positive rates and improve detection rates, which is why AGFEN performs best. In addition to this, we also made a comparison with the latest one-stage detector, the YOLOv5s [38]. As expected, the one-stage method only has an advantage in speed and even lags behind the baseline in detection accuracy, with a gap of up to 7.02% and 8.61% compared with AGFEN in AP and MR^−2^, respectively. Figure 6 is a visual comparison of the two detectors on the CrowdHuman dataset.

### 4.4. State-of-the-Art Comparison on CityPersons Dateset

To further validate the universality of the proposed detector, we also compared it with other representative and advanced detection approaches on the CityPersons dataset, namely Adaptive FasterRCNN [48], OR-CNN [24], Bi-box [27], Repulsion Loss [41], CircleNet [40], FRCN + A + DT [58], FC-Net [19], MGAN+ [14], DMSFLN [16], CSP + HRNet [59], DDAD [60], and ChainDetection + three Modules(CIoU) [61]. The performance of each detector at three commonly used occlusion levels is shown in Table 7. Obviously, DMSFLN and MGAN+ achieved the best scores on the **R** and **HO** sets, respectively. Notably both approaches introduce a visible region branch for learning visible part features and then organically combine the full body branch with the visible body branch. The detector ultimately evaluates the fusion results of the two branches. The difference is that DMSFLN uses cosine similarity to reduce the gap between the two branches, while MGAN+ directly modulates RoI features through spatial attention masks. Therefore, MGAN+ is more direct and effective in dealing with heavy occlusion. Despite the lack of a branch, our AGFEN enhances pedestrian features through two top-down paths and avoids the noise introduced during the fusion of the two branches, making its performance on the **R** set comparable to the first place, only 0.3% behind on the **HO** set, and optimal on the partial set.

Moreover, due to the inclusion of FPN in the detection model, we also conducted a simple comparison and analysis with respect to the capability of small-scale pedestrian detection on the CityPersons dataset. The detectors participating in the comparison are ALFNet [62], CSP [63], and CSP + Offset [63], where +Offset indicates additional offset prediction. The scores in Table 8 demonstrate that our method also has excellent performance in detecting small objects, with improvements of 3.9%, 3.1%, and 1.1% compared with the other three methods.

### 4.5. Comparison with Other Pedestrian Detectors on the Caltech Dataset

Finally, we conducted experiments on Caltech datasets with different occlusion ranges. We have also selected a portion of representative detectors except the previously compared approaches, including DeepParts [25], F-DNN + SS [64], GDFL [65], SDS-RCNN [66], AR Ped [67], etc. From Table 9, it is clear that our AGFEN still exhibits strong competitiveness, achieving MR^−2^ of 6.5%, 36.5%, and 12.9% at three occlusion sets, respectively, without any additional design for occlusion, such as adding a visible region branch. Unconsciously, DMSFLN and MGAN+ once again topped the **R** and **HO** sets, respectively. From the results of the three datasets, it can be seen that an extra visible body branch can indeed improve the performance of detectors, but they must bear extra overhead and design a reasonable fusion scheme. Therefore, in the absence of visible part annotations, our method is a good choice.

## 5. Conclusions

We propose an attention-guided feature enhancement network (AGFEN) for pedestrian detection, with a particular focus on detecting pedestrians in crowded scenes. To achieve this, we introduce an FPN-like structure following the FPN to address its limitations and enhance the texture features of pedestrians. Additionally, a classifier’s weight vector is utilized to aggregate various RoI features, thereby improving RoI feature representation and preventing object confusion. Furthermore, an improved NMS strategy is employed during post-processing to eliminate redundant predictions and reduce the likelihood of suppressing true positives. Finally, we validate the effectiveness of the proposed method on three commonly used datasets: CrowdHuman, CityPersons, and Caltech.

## Figures and Tables

**Table 1:** Representative statistics of three commonly used datasets.

	CrowdHuman	CityPersons	Caltech
images	15,000	2975	42,782
persons	339,565	19,238	13,674
persons/image	22.64	6.47	0.32
unique persons	339,565	19,238	1273

**Table 2:** Ablation study on CrowdHuman validation set. The baseline is ResNet-50 with FPN, and then three modules, AGFP, RoI-A, and OPMP, are embedded in the baseline successively to obtain the scores of different models under the three evaluation metrics. The optimal scores are shown in bold. “✓” indicates that the module is embedded.

Baseline	AGFPN	RoI-A	OPMP	AP/%	MR^−2^/%	JI/%
✓				85.79	42.92	79.83
✓	✓			89.13	41.87	80.94
✓	✓	✓		89.94	41.58	81.27
✓	✓		✓	92.16	41.17	83.12
✓	✓	✓	✓	**92.52**	**40.54**	**83.44**

**Table 3:** Three FPN-like structures are compared on Caltech dataset. The evaluation metric is MR^−2^. The optimal scores are shown in bold.

Model	R	HO
FPN [22]	15.79	82.96
PANet(CircleNet-1/2) [51]	15.80	79.20
CircleNet-1 [40]	13.75	79.87
CircleNet-2 [40]	14.63	75.08
CircleNet-3 [40]	14.72	75.01
AGFPN(CircleNet-1/2)	**10.24**	**66.48**

**Table 4:** A Comparison with other visible-box attention strategies on the CityPersons validation set with MR^−2^. All models use VGG-16 as the backbone. VBB indicates whether a visible region branch is used. The optimal scores are shown in bold.

Method	VBB	Backbone	Scale	R	HO
Bi-box [27]	✓	VGG-16	×1.3	11.2	44.1
OR-CNN [23]	✓	VGG-16	×1.3	11.0	51.3
MGAN [14]	✓	VGG-16	×1.3	**10.3**	49.6
DMSFLN [16]	✓	VGG-16	×1.3	10.7	**44.8**
AGFPN	×	VGG-16	×1.3	10.8	46.5

**Table 5:** Comparison of three NMS approaches and further evaluation of RoI-A on CrowdHuman dataset. The optimal scores are shown in bold.

Method	AP/%	MR^−2^/%	JI/%
NMS	89.13	41.87	80.94
NMS with RoI-A	89.94	41.58	81.27
Soft-NMS	91.53	41.82	80.96
Soft-NMS with RoI-A	91.93	41.46	81.23
OPMP	92.16	41.17	83.12
OPMP with RoI-A	**92.52**	**40.54**	**83.44**

**Table 6:** Score comparison with state-of-the-art detectors on CrowdHuman dataset. The optimal scores are shown in bold.

Model	AP/%	MR^−2^/%	JI/%
Baseline	85.79	42.92	79.83
YOLO v5s [38]	85.50	49.15	-
Adaptive NMS [53]	84.71	49.73	-
JointDet [54]	-	46.50	-
Repulsion Loss [41]	85.64	45.69	-
S-RCNN [52]	90.70	44.70	81.40
D-DETR [55]	91.50	43.70	-
PEDR [56]	91.60	43.70	-
PBM [57]	89.29	43.35	-
DMSFLN [16]	89.18	43.35	-
R^2^NMS [58]	89.29	43.35	-
CSP + HRNet [59]	90.31	43.02	-
DDAD [60]	91.43	43.13	-
ChainDetection + three Modules(CIoU) [61]	91.89	41.67	83.59
AGFEN	**92.52**	**40.54**	**83.44**

**Table 7:** Comparison with the state-of-the-art models on CityPersons validation set with MR^−2^. The optimal scores are shown in bold.

Model	Scale	R	HO	Partial
Adaptive FasterRCNN [48]	×1.3	12.8	-	-
OR-CNN [24]	×1.3	11.0	51.3	13.7
Bi-box [27]	×1.3	11.2	44.15	-
Repulsion Loss [41]	×1.3	11.6	55.3	14.8
CircleNet [40]	×1.3	11.8	50.2	12.2
FRCN + A + DT [58]	×1.3	11.1	44.3	11.2
FC-Net [19]	×1.3	11.6	42.8	11.9
MGAN+ [14]	×1.3	10.3	**37.2**	-
DMSFLN [16]	×1.3	**9.9**	38.1	10.4
CSP + HRNet [59]	×1.3	**10.1**	39.3	10.9
DDAD [60]	×1.3	**10.6**	39.8	11.1
ChainDetection + three Modules(CIoU) [61]	×1.3	**10.3**	37.9	10.6
AGFEN	×1.3	**9.9**	37.5	**10.1**

**Table 8:** Comparison of small-scale pedestrian detection performance on the CityPersons validation set. The evaluation metric is MR^−2^, and the best results are presented in bold.

	ALFNet [62]	CSP [62]	CSP + Offset [63]	AGFEN
Small	19.0	18.2	16.0	**15.1**

**Table 9:** Comparison with the state-of-the-art models on Caltech test set with MR^−2^. The optimal scores are shown in bold.

Model	R	HO	R + HO
DeepParts [25]	11.9	60.4	22.8
ATT-vbb [15]	10.3	45.2	18.2
CircleNet [41]	10.2	44.5	-
Adaptive FasterRCNN [48]	9.2	57.6	20.0
F-DNN + SS [64]	8.2	53.8	18.8
FRCN + A + DT [58]	8.0	37.9	-
GDFL [65]	7.9	43.2	15.6
Bi-box [27]	7.6	44.4	16.1
SDS-RCNN [66]	7.4	58.6	19.7
AR-Ped [67]	6.5	48.8	16.1
DMSFLN [16]	**6.4**	37.5	13.0
MGAN+ [14]	**6.4**	**36.4**	13.2
CSP + HRNet [59]	6.5	36.7	13.4
DDAD [60]	6.5	36.8	13.5
ChainDetection + three Mod-ules(CIoU) [61]	6.5	36.7	13.4
AGFEN	6.5	36.5	**12.9**

## Data Availability

Data are contained within the article.
